# Supplementary material for: COVID-19 vaccine hesitancy among Ethiopian healthcare workers
Source: PLoS One. 2021 Dec 17;16(12):e0261125. doi: 10.1371/journal.pone.0261125 (PMC8682893; doi:10.1371/journal.pone.0261125)
Supplement: S1 File — (DOCX) [file pone.0261125.s001.docx]

**SUPPLEMENTARY FILE**

**Part 1: information sheet**

Greetings; I am ………………… and I am here to obtain information for the study which is being conducted in Addis Ababa public Hospitals among healthcare providers.

You will proceed to fill this questionnaire which will take around 30 minutes, after hearing the following general information about the study.

**Title of the study:** COVID-19 vaccine hesitancy among health care providers working in hospital in Addis Ababa, Ethiopia 2021.

**Objective:** To assess COVID-19 vaccine hesitancy among health care providers working in hospitals in Addis Ababa, Ethiopia 2021.

**Benefit:** This study will not give any direct benefit to the participants; but the information and the samples which will be obtained from you will help the researchers to recommend the government and other concerned bodies to design appropriate intervention to address the problems.

**Risk:** The study will not impose any risk on the participants.

**Right of the respondents:** Any participant will participate on this study voluntarily. You can quit from giving answers to the questions you are not willing to answer and even you can stop responding at all.

**Confidentiality:** The completed questionnaires will not be accessed by anyone other than the study team and any information that you will give will be confidential.

**Whom to contact:** If you will have any questions about the research or need further information please contact Rihanna Mohammed, at Africa medical college department of master of public health, using phone number +251941228029 or Email- [mohammedrihanna3@gmail.com](mailto:mohammedrihanna3@gmail.com). If you have any questions about the content and purpose of the study, you can ask me. Are you willing to participate?

**Consent form**

I, ____________________ hereby give my consent to participate in this study. I have been given the necessary information about the research in a language I understand. I have also understood that I can withdraw my consent any time without penalty of loss of benefits.

1. If yes, proceed to the next page
2. If no ,thank you and skip to the next participant

- Name of the data collector:_____________________ signature: ___________
- Name of supervisor: _____________________ signature:_______________
- Date of interview:_______________________

**Part 2: Semi-structured questionnaire**

**Questionnaire for the assessment of COVID-19 vaccine hesitancy among health care providers of public Hospitals in Addis Ababa, Ethiopia 2021.**

Serial No.:_____________________

**Name of Hospital you are currently working at__________________**

| **Section 1: Exposure to COVID-19 infection** | | | | | | | | | | | | | | | | | | | | | | | | | | | | | | | | |
| --- | --- | --- | --- | --- | --- | --- | --- | --- | --- | --- | --- | --- | --- | --- | --- | --- | --- | --- | --- | --- | --- | --- | --- | --- | --- | --- | --- | --- | --- | --- | --- | --- |
| 101 | | Have you been previously in contact with COVID-19 infected patients? | | | | | | | | | | | | | | | 1. Yes | | | | | | | | | 1. No | | | | | 1. Not sure | |
| 102 | | Have you been previously in contact with COVID-19 positive family member or friend? | | | | | | | | | | | | | | | 1. Yes | | | | | | | | | 1. No | | | | | 1. Not sure | |
| 103 | | Have you been taking care of and/or treating COVID-19 patients? | | | | | | | | | | | | | | | 1. Yes | | | | | | | | | 1. No | | | | |  | |
| 104 | | Have you been infected with laboratory confirmed COVID-19 disease? | | | | | | | | | | | | | | | 1. Yes | | | | | | | | | 1. No | | | | |  | |
| **Section 2. Knowledge and attitude towards COVID-19 vaccine** | | | | | | | | | | | | | | | | | | | | | | | | | | | | | | | | |
| **Code** | | **Question** | | | | | | | | | **Response** | | | | | | | | | | | | | | | | | | | | | |
| 201 | | Did you take training on COVID-19? | | | | | | | | | 1. Yes 2. No | | | | | | | | | | | | | | | | | | | | | |
| 202 | | Are you willing to be vaccinated with the COVID-19 vaccine when it becomes accessible? | | | | | | | | | 1. Yes, certainly 2. Yes, probably 3. No, probably not 4. No, certainly not 5. Do not know | | | | | | | | | | | | | | | | | | | | | |
| 203 | | When will you take COVID-19 vaccine? | | | | | | | | | 1. I will take a shot as soon as possible 2. I will delay getting it for few months 3. I will never take the vaccine | | | | | | | | | | | | | | | | | | | | | |
| 204 | | If a vaccine against COVID-19 is accessible to the public would you be willing to recommend it to your patients/clients and other community members? | | | | | | | | | 1. Yes, certainly 2. Yes, probably 3. No, probably not 4. No, certainly not 5. Do not know | | | | | | | | | | | | | | | | | | | | | |
| 205 | | What is your belief about COVID-19 vaccines? | | | | | | | | | 1. COVID-19 vaccine is the most likely way to stop this pandemic 2. The best way to avoid the complications of COVID-19 is being vaccinated 3. COVID-19 vaccines are harm full and useless | | | | | | | | | | | | | | | | | | | | | |
| 206 | | It is preferable to acquire immunity against infectious disease naturally (by having the disease) than by vaccination. | | | | | | | | | 1. Agree 2. Disagree 3. Do not know | | | | | | | | | | | | | | | | | | | | | |
| 207 | | What is/are your best sources of information about COVID-19 and its vaccines? You can choose more than one answer | | | | | | | | | 1. Social media 2. The government 3. Television/Radio 4. Tele communication 5. Peer 6. Religious place 7. Others, specify________________________ | | | | | | | | | | | | | | | | | | | | | |
| 208 | | Have you ever received any vaccine at your adult age? | | | | | | | | | 1. Yes | | | | | | | | | | | | | 1. No | | | | | | 1. Not sure | | |
| 209 | | The COVID-19 vaccine contains live viruses that may cause some people to get COVID-19 | | | | | | | | | 1. Agree | | | | | | | | | | | | | 1. Disagree | | | | | | 1. Unsure | | |
| 2010 | | Getting vaccinated against COVID-19 is important to protect patients | | | | | | | | | 1. Agree | | | | | | | | | | | | | 1. Disagree | | | | | | 1. Unsure | | |
| 2011 | | Which factor most influenced you to take or not to take the COVID-19 vaccine? Choose only one. | | | | | | | | | 1. Religious views 2. Information or advice from peers 3. Information from vaccine producers 4. Information from media(Electronic such as TV, Radio, social media and printed media’s) 5. Others, specify___________ | | | | | | | | | | | | | | | | | | | | | |
| **Section 3: Perceived concerns of health care providers regarding COVID vaccine** | | | | | | | | | | | | | | | | | | | | | | | | | | | | | | | | |
| 301 | | The currently available COVID-19 vaccines are safe | | | | | | | | | | 1. Agree | | | | | | | | | | 1. Disagree | | | | | | | 1. Unsure | | | |
| 302 | | The currently available COVID-19 vaccines are effective | | | | | | | | | | 1. Agree | | | | | | | | | | 1. Disagree | | | | | | | 1. Unsure | | | |
| 302 | | Are you concerned about the safety of the COVID-19 vaccine? | | | | | | | | | | 1. Yes 2. No 3. I need more information on the safety of COVID-19 vaccine before a decision is made as to whether to receive it or not. | | | | | | | | | | | | | | | | | | | | |
| 303 | | Are you concerned about the efficacy of the COVID-19 vaccine? | | | | | | | | | | 1. Yes 2. No 3. I need more information on the efficacy of COVID-19 vaccine before a decision is made as to whether to receive it. | | | | | | | | | | | | | | | | | | | | |
| 304 | | If your answer for question #301 and 302 is yes, what kind of risks are you concerned about? You can answer more than 1 | | | | | | | | | | 1. It may not provide short and long term protection 2. The potential short term and long term side-effects 3. Risk of COVID-19 infection due to COVID-19 vaccine it self 4. Others___________________________________________________________________________________________________________________ | | | | | | | | | | | | | | | | | | | | |
| 305 | | The side effects of COVID-19 vaccine are not acceptable to me | | | | | | | | | | 1. Agree | | | | | | | | 1. Disagree | | | | | | | | 1. Unsure | | | | |
| 306 | | I don’t believe that COVID-19 immunization will benefit me because I have good immunity and I won’t get COVID-19 | | | | | | | | | | 1. Agree | | | | | | | | 1. Disagree | | | | | | | | 1. Unsure | | | | |
| 306 | | Are you concerned about any risk with the vaccine? | | | | | | | | | | 1. Yes | | | | | | | | 1. No | | | | | | | | 1. Do not know | | | | |
| 307 | | Do you believe vaccines are safe for yourself, for your child/children, for those in your community? | | | | | | | | | | 1. Yes | | | | | | | | 1. No | | | | | | | | 1. Not sure | | | | |
| 308 | | I have a medical contradiction to COVID-19 vaccine | | | | | | | | | | 1. Yes | | | | | | | | 1. No | | | | | | | | 1. Unsure | | | | |
| 309 | | Do you trust science to develop safe and effective vaccines? | | | | | | | | | | 1. Yes | | | | | | | | 1. No | | | | | | | | 1. Not sure | | | | |
| 3010 | | Do you trust the ministry of health to ensure that COVID-19 vaccine is safe? | | | | | | | | | | 1. Yes | | | | | | | | 1. No | | | | | | | | 1. Not sure | | | | |
| **Section 4: Healthcare providers perceived worries from COVID-19 infection** | | | | | | | | | | | | | | | | | | | | | | | | | | | | | | | | |
| 401 | How worried are you about your personal health due to your role in the hospital during COVID-19 pandemic? | | | | | | | | | | 1. Extremely worried 2. Somewhat worried 3. Not worried at all | | | | | | | | | | | | | | | | | | | | | |
| 402 | How worried are you about the potential risk of becoming infected with COVID-19 due to your role in the hospital? | | | | | | | | | | 1. Extremely worried 2. Somewhat worried 3. Not worried at all | | | | | | | | | | | | | | | | | | | | | |
| 403 | How worried are you about the potential risk of COVID-19 to your family, loved ones or others due to your role in the hospital? | | | | | | | | | | 1. Extremely worried 2. Somewhat worried 3. Not worried at all | | | | | | | | | | | | | | | | | | | | | |
| **Section 5: Socio-demographic characteristics** | | | | | | | | | | | | | | | | | | | | | | | | | | | | | | | | |
| 501 | | Age | | ……………years | | | | | | | | | | | | | | | | | | | | | | | | | | | | |
| 502 | | Sex | | 1. Male | | | 1. Female | | | | | | | | | | | | | | | | | |  | |  | | | | | |
| 503 | | Marital status | | 1. Married | | | 1. Single | | | | | | 1. Divorced | | | | | | | | | | | | | | 1. Widowed | | | | | |
| 504 | | Religion | 1. Orthodox Christian | | | 1. Muslim | | | | 1. Protestant Christian | | | | | | | | 1. Catholic Christian | | | | | | | | | 1. Other (specify)____________ | | | | | |
| 505 | | Profession | 1. Medical Doctor | | 1. Nurse | | | 1. Midwives | | | | | | | 1. Lab | | | | | | 1. HO | | | | | 1. Pharmacy | | | | | | Other, specify…… |
| 506 | | Level of education in health science | | | 1. Diploma | | | | 1. BSc degree | | | | | | | 1. MD | | | | | | | 1. Specialist | | | | | | 1. Masters and above | | | |
| 507 | | Years of experience as a healthcare professional | | | ………………….years | | | | | | | | | | | | | | | | | | | | | | | | | | | |
|  | | Primary work area or unit | | | 1. COVID-19 management wing 2. Adult medical ward 3. Pediatric medical ward 4. Adult ICU 5. Pediatric ICU 6. NICU 7. Adult or pediatric surgical ward | | | | | | | | | | | | | | 1. Gyn/Obs 2. Emergency 3. Oncology 4. Rotation (among different wards) 5. Pharmacy 6. Laboratory 7. Others, please specify _____________________ | | | | | | | | | | | | | |
| 508 | | Do you have confirmed chronic illness? | | | 1. Yes | | | | | | | | | 1. No | | | | | | | | | | | | | | | | | | |
| 509 | | If you have confirmed chronic medical condition, can you specify | | | 1. Hypertension 2. DM 3. CHF 4. RVI | | | | | | | | | 1. Psychiatric illnesses 2. Hypertension + DM 3. CHF + Hypertension 4. CHF + Hypertension + DM 5. Others, specify ____________________________ | | | | | | | | | | | | | | | | | | |
